# Validity of Short and Long Self-Administered Food Frequency Questionnaires in Ranking Dietary Intake in Middle-Aged and Elderly Japanese in the Japan Public Health Center-Based Prospective Study for the Next Generation (JPHC-NEXT) Protocol Area

**DOI:** 10.2188/jea.JE20150064

**Published:** 2016-08-05

**Authors:** Yuta Yokoyama, Ribeka Takachi, Junko Ishihara, Yuri Ishii, Shizuka Sasazuki, Norie Sawada, Yurie Shinozawa, Junta Tanaka, Erika Kato, Kaori Kitamura, Kazutoshi Nakamura, Shoichiro Tsugane

**Affiliations:** 1 Department of Community Preventive Medicine, Niigata University Graduate School of Medical and Dental Sciences, Niigata, Japan; 1 新潟大学大学院医歯学総合研究科環境予防医学分野; 2 Department of Food Science and Nutrition, Faculty of Human Life and Environment, Nara Women’s University, Nara, Japan; 2 奈良女子大学研究院生活環境科学系食物栄養学領域; 3 Department of Nutrition Science, Sagami Women’s University, Sagamihara, Kanagawa, Japan; 3 相模女子大学栄養科学部管理栄養学科; 4 Epidemiology and Prevention Group, Research Center for Cancer Prevention and Screening, National Cancer Center, Tokyo, Japan; 4 国立がん研究センターがん予防・検診研究センター予防研究グループ; 5 Department of Health Promotion Medicine, Niigata University Graduate School of Medical and Dental Sciences, Niigata, Japan; 5 新潟大学大学院医歯学総合研究科健康増進医学講座

**Keywords:** validity, food frequency questionnaire, food records, Japanese, 妥当性, 食物摂取頻度調査, 食事記録, 日本人

## Abstract

**Background:**

Longitudinal epidemiological studies require both the periodic update of intake information via repeated dietary survey and the minimization of subject burden in responding to questionnaires. We developed a 66-item Food Frequency Questionnaire (short-FFQ) for the Japan Public Health Center-based prospective Study for the Next Generation (JPHC-NEXT) follow-up survey using major foods from the FFQ developed for the original JPHC Study. For the JPHC-NEXT baseline survey, we used a larger 172-item FFQ (long-FFQ), which was also derived from the JPHC-FFQ. We compared the validity of ranking individuals by levels of dietary consumption by these FFQs among residents of selected JPHC-NEXT study areas.

**Methods:**

From 2012 to 2013, 240 men and women aged 40–74 years from five areas in the JPHC-NEXT protocol were asked to respond to the long-FFQ and provide 12-day weighed food records (WFR) as reference; 228 also completed the short-FFQ. Spearman’s correlation coefficients (CCs) between estimates from the FFQs and WFR were calculated and corrected for intra-individual variation of the WFR.

**Results:**

Median CC values for energy and 53 nutrients for the short-FFQ for men and women were 0.46 and 0.44, respectively. Respective values for the long-FFQ were 0.50 and 0.43. Compared with the long-FFQ, cross-classification into exact plus adjacent quintiles with the short-FFQ ranged from 68% to 91% in men and 58% to 85% in women.

**Conclusions:**

Similar to the long-FFQ, the short-FFQ provided reasonably valid measures for ranking middle-aged and elderly Japanese for many nutrients and food groups. The short-FFQ can be used in follow-up surveys in prospective cohort studies aimed at updating diet rank information.

## Abstract (ja)

**背景・目的：:**

長期の前向きコホート研究では、追跡調査による摂取量情報の更新と同時に、被調査者の回答にかかる負担軽減も同時に要求される。我々は、次世代多目的コホート研究（JPHC-NEXT）ベースライン調査で、JPHC Study FFQを基に開発した詳細版FFQ（172食品項目）を用いたが、追跡調査のために短縮版FFQ（66食品項目）を開発した。そこで、ベースラインで用いた短縮版及び詳細版FFQの順位づけ妥当性を当該調査対象の地域住民にて比較した。

**方法：:**

2012年～2013年に、JPHC-NEXT 詳細版FFQを使用する国内５地域の40–74歳の男女240人が12日間の秤量食事記録調査(12d-WFR)、詳細版FFQへ回答を完了した。そのうち228 人は短縮版FFQも提出した。２種類のFFQから計算した摂取量推定値と12d-WFRとのSpearman相関係数を求め、個人内変動を補正した。

**結果：:**

短縮版FFQによるエネルギー及び53栄養素項目の推定値と12d-WFRとの相関係数の中央値は、男性で0.46、女性で0.44であった。詳細版のそれはそれぞれ0.50、0.43であった。短縮版・詳細版FFQによる栄養素等摂取量５分位による順位づけの、同分位か隣接分位内に一致した者の割合は、男性で68%～91%、女性では58%～85%であった。

**まとめ：:**

中高年の地域住民において、短縮版FFQでも、詳細版FFQと同様に多くの栄養素・食品群で概ね中程度以上に順位づけ可能な推定値が得られることが確認された。短縮版FFQは、前向き研究の追跡調査において食事情報の更新を目的として利用可能である。

## INTRODUCTION

An accurate understanding of habitual dietary intake is an essential component of many epidemiological studies. One of the most frequently used methods of determining habitual dietary intake is the food frequency questionnaire (FFQ). Because FFQs are simple to implement in large-scale studies and allow the ranking of individuals by estimated dietary intake, they are widely used to evaluate diet-disease associations, subject to verification of the accuracy of the intake estimates they provide.^1^ The food list used in an FFQ must be suitable for the dietary culture and habits of the study population,^2^ and the validity of FFQ estimates of dietary intake appears to depend on the specific population.^1^

Because the Japanese dietary model involves consumption of varied combinations of many foods, development of a FFQ food list according to the percentage contribution to absolute intake requires inclusion of a large number of items. However, increasing the number of items does not remarkably improve validity^3^ and may conversely result in an increase in the number of subjects who drop out or an increase in the number of items with missing data. Longitudinal epidemiological studies need to reduce subject burden in responding to the questionnaire. Moreover, evaluation of the association between dietary habits and disease over a long period in prospective cohort studies is aided by periodic update of intake information.^1^

These background factors require that an FFQ used in an epidemiological study be examined for its validity for the specific study subjects and region. The FFQ should also include a minimum food list, which enables intake to be estimated efficiently, and must be evaluated for its suitability in updating the intake ranking.

One objective of this study was to verify the validity of estimates obtained with a short-list version of the FFQ (short-FFQ) developed for the Japan Public Health Center-based prospective Study for the Next Generation (JPHC-NEXT) follow-up survey. The JPHC-NEXT project is a molecular epidemiological cohort study investigating the associations between lifestyle, including dietary habit, and various non-communicable diseases or failures, as well as their genetic interaction effect.^4^ We compared short-FFQ-derived estimates with dietary intake based on a 12-day weighed food record (12d-WFR) of middle-aged and elderly residents in the JPHC-NEXT protocol areas. The other objective was to compare the validity of the short-FFQ estimates with those obtained using the full version of the FFQ (long-FFQ), which consists of 172 foods used in the baseline survey of the JPHC-NEXT protocol. Few simultaneous validation studies have compared estimates obtained with the long and short versions of an FFQ,^5^^,^^6^ and no such study has been aimed at updating information on dietary intake in a longitudinal cohort study. In addition, to validate the use of the short-FFQ for intake updates in the prospective study, we also examined concordance between the rankings of intake obtained with the different versions of the FFQ.

## METHODS

### Study settings and participants

The study was conducted in five areas included in the protocol for the JPHC-NEXT (Yokote, Saku, Chikusei, Murakami, and Uonuma).^4^ Subjects were middle-aged and elderly residents of these five areas. Through recruitment by the cohort-study office in each area, 255 generally healthy men and women participated in this study on a voluntary basis. Sample size was calculated to allow detection of a correlation coefficient of 0.25,^7^ which was observed in a previous study for vitamin A having the largest within-person variation.^1^ The study was approved by the Institutional Review Board of the National Cancer Center, Tokyo, Japan and all other collaborating research institutions. All participants provided written informed consent to participate at the study orientation. Of the 253 participants who completed the surveys, 240 subjects (98 men and 142 women) aged 40–74 years at the start of this validation study were defined as the study subjects.

### Data collection

**Figure 1.**
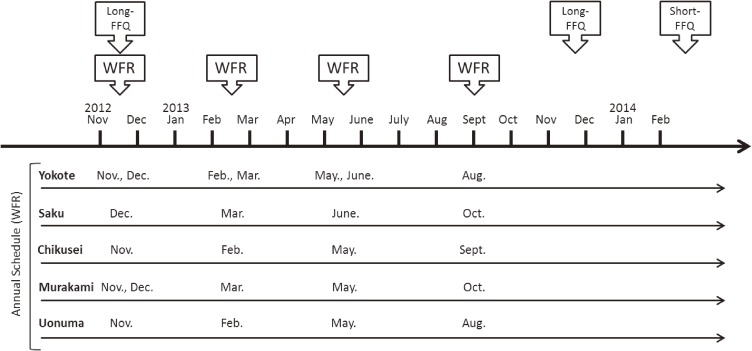
Data collection sequence in the Short- and Long-FFQs Validation Study. Long-FFQ, 172-item food frequency questionnaire; Short-FFQ, 66-item food frequency questionnaire; WFR, consecutive 3-day weighed food record.

Between November 2012 and December 2013, reference intake data were obtained from all participants using the 3-consecutive-day weighed food records over four seasons (12d-WFR) at intervals of approximately 3 months (Figure 1).

The self-administered long-FFQ for the JPHC-NEXT protocol was administered twice at an interval of 1 year. Information on height, weight, and smoking and drinking habits were collected using self-report questionnaire integrated with the second long-FFQ. To determine the validity of estimated dietary intake based on this long-FFQ, we used information from the second administration because the FFQ asks about the diet of the past year.

Of the 240 subjects, 92 men and 136 women were also provided with the short-FFQ in February 2014.

### Reference methods

The 12d-WFR consisted of two weekdays and one weekend day in each of the four seasons. Food portions were measured by each participant during meal preparation using a supplied portable precise digital cooking scale (Tanita Co. Ltd, Tokyo, Japan) and measuring spoons and cups. For foods purchased or consumed outside the home, the participants were instructed to record the approximate quantity of all foods in the meal and/or the name of the product and company. Food records were checked by trained dietitians with the participants the day after each of the 3-consecutive-day WFRs on site in each study area, and the foods and weights were coded. In some cases, the 3-day WFR was submitted by fax or mail to the study office and checked with the subject over the telephone.

### Long- and short-FFQs

The long-FFQ consisted of 172 food and beverage items and nine frequency categories, ranging from almost never to seven or more times per day (or to 10 or more glasses per day, for beverages). It asked about the usual consumption of listed foods during the previous year. The food list was initially developed according to percentage contributions based on absolute values of energy and intake of 14 target nutrients from weighed food records in 1989–1991^8^ and used for the Japan Public Health Center-based prospective Study,^8^^–^^12^ for which it was modified for middle-aged and elderly residents in a wide variety of areas of Japan. With regard to this modification, the following criteria were considered: calculation for an additional 17 nutrient items, such as fiber and folate, change of foods contributing to the absolute nutrient intake according to the updated Standard Tables of Food Composition in Japan,^13^^,^^14^ and dietary regionality and change in generation for the present cohort (data not shown). As a result, 33 foods were added, and 5 foods and beverages were excluded.^15^ Moreover, six foods were also added to account for potential inter-individual variation in specific nutrients, such as isothiocyanate and isoflavone. With regard to alcoholic beverages, choices of intake amount were changed from the initial JPHC-FFQ.

To develop the food list for the short-FFQ, we selected and combined items and supporting questions from the original long-FFQ. We selected the three major foods and beverages that contributed to inter-individual variation for each of 40 nutrients according to a cumulative *R*^2^ for the 40 nutrients,^16^ based on the multiple regression coefficient with total intake of a specific nutrient as the dependent variable and its intake from each food as the explanatory variable. Inter-individual variation was calculated by gender among 45 869 men and 52 989 women who responded to the JPHC Study 10-year follow-up survey. Consequently, cumulative *R*^2^ for the nutrients ranged from 0.4 to 1.0. For potential inter-individual variation in intake of specific food groups, some foods, such as coffee, were added. Ultimately, 66 food and beverage items were selected for the short-FFQ. In this validation study, information on alcoholic beverages was substituted with those from the long-FFQ (united with overall information of lifestyle), because these questions were not included in the short-FFQ. This was because information on alcoholic beverage intake was structured in pages for lifestyle other than diet, such as smoking status and physical activity, and the reproducibility of alcoholic beverage intake was relatively high even if questionnaires were administered at a 1-year interval.^17^^,^^18^

Intakes of energy, 53 nutrients, and 29 food groups were calculated using the Standard Tables of Food Composition in Japan 2010,^19^ Standard Tables of Food Composition in Japan Fifth Revised and Enlarged Edition 2005 For Fatty Acids,^20^ and a specifically developed food composition table for isoflavones in Japanese foods.^21^

### Statistical analysis

**Figure 2.**
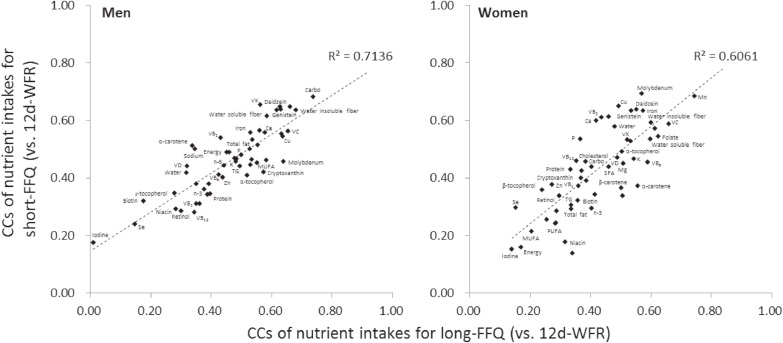
Scatter plot between CCs of the short-FFQ and those of the long-FFQ (vs 12-day weighed food record for both) for men and women. X-axis: CCs of nutrient intakes assessed by the long-FFQ (vs 12-day weighed food record); Y-axis: CCs of nutrient intakes assessed by the short-FFQ (vs 12-day weighed food record) CC, correlation coefficient; Long-FFQ, 172-item food frequency questionnaire; Short-FFQ, 66-item food frequency questionnaire; WFR, consecutive 3-day weighed food record.

The mean intakes of each nutrient and food group, estimated using the long- and short-FFQs, were compared to intakes estimated using the 12d-WFR among 98 men and 142 women for the long-FFQ and 92 men and 136 women for the short-FFQ. Percentage differences were calculated for each nutrient and food group by dividing the difference in intake in the long- and short-FFQs from that in the 12d-WFR. To determine the validity of the long- and short-FFQs, Spearman’s rank correlation coefficients (CCs) between intakes based on the FFQs and 12d-WFR were calculated for energy-adjusted values. A residual model was used for energy adjustment.^1^ We corrected the observed CCs for the attenuating effect of random intra-individual error from the usual intake of each energy, nutrient, and food group.^1^^,^^15^ CCs for estimates of nutrients using the short- and long-FFQ compared to the 12d-WFR are shown as a scatter plot in Figure 2. To compare the agreement of categorization of estimated intake based on the short-FFQ with that of the 12d-WFR or long-FFQ, we compared the number of participants classified into the same, adjacent, and extreme categories by cross-classification according to quintile. All analyses were performed using SAS Version 9.4 (SAS Institute Inc., Cary, NC, USA).

## RESULTS

Subjects who completed the long-FFQ are characterized in Table 1. Mean age was 57.4 years in men and 57.0 years in women. Mean (standard deviation) body mass index (BMI) was 23.7 (2.8) in men and 22.8 (3.1) in women. The proportion of current smokers and heavy drinkers was 26.5% and 39.8% among men and 2.1% and 4.9% among women, respectively. These characteristics did not differ from those of respondents to the short-FFQ only (92 men and 136 women).

**Table 1.** Characteristics of subjects who completed the long-FFQ (98 men and 142 women)

	Men	Women
Age, years^a^	57.4 (8.6)	57.0 (8.6)
Body height, cm^a^	168.2 (6.8)	156.6 (5.7)**
Body weight, kg^a^	67.0 (9.3)	55.9 (8.0)**
BMI, kg/m^2 a^	23.7 (2.8)	22.8 (3.1)*
Current smoker, %	26.5%	2.1%
Heavy drinker,^b^ %	39.8%	4.9%

BMI, body mass index; FFQ, Food Frequency Questionnaire.

^a^Values are reported as mean (standard deviation).

^b^≥280 g ethanol/wk in men and ≥140 g ethanol/wk in women. *P*-values refer to student’s *t*-test between sex for each; *P < 0.05, **P < 0.01.

### Validity of the long- and short-FFQ for mean intakes

Table 2 and Table 3 show daily intake of energy and 53 nutrients as assessed by the 12d-WFR and long- and short-FFQs; percentage differences between each FFQ and the 12d-WFR; and their correlations for men and women. Percentage differences in energy intake based on the long- and short-FFQs with the 12d-WFR varied from +3% to −21% in men and +13% to −24% in women. Almost all of the nutrient intakes based on the short-FFQ were underestimated compared with those of the 12d-WFR and the long-FFQ in men and women. The CCs of total energy intake for both FFQs were similar, although they were lower in women than in men. The median (range) values across energy and deattenuated CCs of energy-adjusted nutrient intakes based on the long- and short-FFQs were 0.50 (0.01–0.82) and 0.46 (0.18–0.68) in men and 0.43 (0.14–0.74) and 0.44 (0.15–0.69) in women. These CCs for the short-FFQ were similar to those for the long-FFQ among both men and women. Figure 2 shows the scatter plot of these CCs for intake of energy and each nutrient for the short-FFQ (vs the 12d-WFR) and those for the long-FFQ, and the Pearson’s CCs were 0.7 in men and 0.6 in women.

**Table 2.** Energy and nutrient intakes according to long-FFQ/short-FFQ, percentage differences between intakes by two FFQs and 12d-WFR and their correlations in men

	Men											

	Long-FFQ (*n* = 98)						Short-FFQ (*n* = 92)					
	
	12d-WFR		FFQ		%^a^	CC^b,c^	12d-WFR		FFQ		%^a^	CC^b,c^
			
	Mean	(SD)	Mean	(SD)			Mean	(SD)	Mean	(SD)		
Energy, kcal	2315	(447)	2390	(697)	3	0.45^c^**	2345	(440)	1857	(517)	−21**	0.49^c^**
Water, g	2683	(644)	2915	(1089)	9*	0.32**	2726	(615)	2311	(924)	−15**	0.42**
Protein, g	83.7	(18.1)	78.8	(31.8)	−6	0.40**	84.8	(17.6)	60.9	(24.3)	−28**	0.35**
Sum of amino acid residues	29.4	(7.1)	31.1	(11.0)	6	0.36**	29.9	(7.1)	26.4	(11.3)	−12**	0.31**
Total fat, g	62.6	(16.9)	61.1	(29.3)	−2	0.53**	63.9	(16.4)	42.3	(22.5)	−34**	0.50**

Total fat in % energy	24.2	(3.9)	22.5	(6.1)	−7**	0.46^c^**	24.5	(3.8)	19.9	(6.9)	−18**	0.49^c^**
Saturated fatty acid, g	17.1	(5.4)	17.5	(9.3)	3	0.48**	17.4	(5.3)	12.1	(7.0)	−30**	0.47**
Monounsaturated fatty acid, g	22.8	(6.5)	22.6	(11.1)	−1	0.55**	23.3	(6.3)	15.4	(8.4)	−34**	0.45**
Polyunsaturated fatty acid, g	13.6	(3.6)	13.7	(6.8)	1	0.50**	13.9	(3.4)	9.4	(5.1)	−32**	0.48**
n-3 PUFA	2.9	(0.9)	2.6	(1.4)	−11*	0.38**	3.0	(0.9)	2.0	(1.1)	−33**	0.36**
n-6 PUFA	10.5	(2.9)	11.0	(5.6)	5	0.44**	10.7	(2.8)	7.4	(4.1)	−31**	0.44**
Triacylglycerol equivalents, g	54.8	(15.0)	56.1	(26.9)	2	0.49**	56.0	(14.5)	38.5	(20.6)	−31**	0.44**
Cholesterol, mg	369.4	(116.6)	341.1	(341.3)	−8	0.53**	373.7	(114.7)	293.8	(291.3)	−21**	0.45**

Carbohydrate, g	300.1	(63.1)	309.7	(100.5)	3	0.74**	302.3	(63.8)	235.6	(72.3)	−22**	0.68**

Total dietary fiber, g	16.8	(5.7)	14.5	(7.0)	−14**	0.66**	17.0	(5.7)	9.0	(4.4)	−47**	0.65**
Water soluble fiber, g	3.7	(1.3)	3.4	(1.8)	−9*	0.58**	3.8	(1.3)	1.9	(1.2)	−49**	0.62**
Water insoluble fiber, g	12.4	(4.3)	10.6	(5.1)	−14**	0.68**	12.6	(4.3)	6.7	(3.2)	−46**	0.64**

Sodium, mg	4570	(1092)	4360	(2077)	−5	0.34**	4622	(1060)	2958	(1439)	−36**	0.50**
Potassium, mg	3105	(887)	3142	(1298)	1	0.48**	3150	(860)	2177	(923)	−31**	0.46**
Calcium, mg	570	(182)	595	(393)	4	0.58**	577	(177)	362	(251)	−37**	0.56**
Magnesium, mg	325	(86)	353	(130)	9*	0.39**	329	(84)	266	(106)	−19**	0.34**
Phosphorus, mg	1258	(290)	1247	(514)	−1	0.48**	1275	(282)	922	(368)	−28**	0.47**
Iron, mg	9.5	(2.5)	9.7	(4.1)	2	0.53**	9.7	(2.4)	7.6	(3.3)	−21**	0.56**
Zinc, mg	9.6	(2.3)	9.3	(3.2)	−3	0.44**	9.7	(2.3)	7.2	(2.5)	−26**	0.40**
Copper, mg	1.44	(0.36)	1.40	(0.50)	−2	0.64**	1.46	(0.35)	1.05	(0.38)	−28**	0.54**
Manganese, mg	4.53	(1.55)	4.50	(1.94)	−1	0.58**	4.61	(1.56)	3.56	(1.70)	−23**	0.46**
Iodine, µg	1934	(3976)	202	(192)	−90**	0.01	1668	(2896)	153	(155)	−91**	0.18
Selenium, µg	61	(19)	66	(31)	9	0.15	61	(19)	47	(25)	−23**	0.24*
Chromium, µg	8	(3)	7	(4)	−10	0.39**	8	(3)	4	(3)	−45**	0.38**
Molybdenum, µg	216	(68)	249	(84)	15**	0.64**	218	(68)	233	(89)	7	0.46**

Retinol, µg	267	(346)	342	(359)	28	0.30**	276	(354)	438	(519)	59**	0.29**
Alpha-carotene, µg	498	(295)	458	(449)	−8	0.34**	509	(301)	421	(405)	−17*	0.51**
Beta-carotene, µg	3649	(1703)	3022	(2377)	−17**	0.55**	3719	(1718)	2442	(2086)	−34**	0.51**
Cryptoxanthin, µg	315	(348)	607	(544)	93**	0.57**	320	(356)	688	(970)	115**	0.42**
Beta carotene equivalents, µg	4263	(1975)	3562	(2671)	−16**	0.53**	4339	(1983)	2987	(2470)	−31**	0.46**
Retinol equivalents, µg	639	(380)	642	(468)	1	0.35**	653	(382)	691	(613)	6	0.38**
Vitamin D, µg	11.3	(5.2)	9.1	(5.9)	−20**	0.32**	11.5	(5.2)	8.1	(5.6)	−29**	0.44**
Alpha-tocopherol, mg	8.5	(2.6)	8.0	(4.0)	−5	0.52**	8.7	(2.5)	4.8	(2.6)	−44**	0.41**
Beta-tocopherol, mg	0.4	(0.1)	0.4	(0.2)	10	0.54**	0.4	(0.1)	0.3	(0.2)	−32**	0.53**
Gamma-tocopherol, mg	11.1	(3.3)	11.1	(6.1)	0	0.28**	11.4	(3.2)	7.7	(5.2)	−32**	0.35**
Delta-tocopherol, mg	2.9	(1.0)	2.6	(1.9)	−8	0.56**	2.9	(1.0)	2.2	(1.7)	−23**	0.57**
Vitamin K, µg	298	(132)	270	(163)	−9	0.56**	303	(132)	207	(156)	−31**	0.65**
Vitamin B_1_, mg	1.26	(0.52)	1.02	(0.40)	−19**	0.35**	1.29	(0.52)	0.80	(0.37)	−38**	0.31**
Vitamin B_2_, mg	1.68	(0.62)	1.46	(0.77)	−13*	0.43**	1.71	(0.62)	1.14	(0.59)	−34**	0.54**
Niacin, mg	23.7	(6.9)	24.9	(9.8)	5	0.28**	24.1	(6.8)	20.3	(8.9)	−16**	0.29**
Vitamin B_6_, mg	1.84	(0.91)	1.64	(0.63)	−11*	0.42**	1.88	(0.92)	1.34	(0.56)	−29**	0.41**
Vitamin B_12_, µg	9.8	(4.3)	7.7	(4.5)	−21**	0.34**	9.9	(4.3)	7.2	(4.6)	−28**	0.28**
Folate, µg	453	(159)	403	(204)	−11**	0.63**	461	(158)	291	(168)	−37**	0.55**
Pantothenic acid, mg	7.16	(1.84)	7.63	(3.21)	7	0.62**	7.28	(1.80)	5.81	(2.33)	−20**	0.64**
Biotin, µg	35.0	(10.3)	44.0	(18.2)	26**	0.17	35.6	(10.1)	37.3	(17.4)	5	0.32**
Vitamin C, mg	142	(71)	123	(86)	−13**	0.65**	145	(71)	65	(54)	−55**	0.56**

Daidzein, mg	13.92	(8.51)	17.12	(16.62)	23*	0.63**	14.29	(8.62)	17.07	(18.48)	19	0.65**
Genistein, mg	23.41	(14.31)	28.16	(27.89)	20*	0.63**	24.02	(14.50)	27.47	(29.97)	14	0.64**

Ethanol, g	27.4	(24.9)	35.1	(30.7)	28**	0.82**	28.2	(25.4)	NA	NA	NA	NA

**MEDIAN**						**0.50**						**0.46**

12d-WFR, 12-day weighed food records; CC, correlation coefficient; FFQ, Food Frequency Questionnaire; PUFA, poly-unsaturated fatty acids; NA, not applicable for calculation; SD, standard deviation.

^a^Percentage differences: (FFQ-12d-WFR)/12d-WFR × 100 (%). *P*-values refer to paired *t*-test between intakes by each FFQs and those by 12d-WFR for each; *P < 0.05, **P < 0.01.

^b^Spearman’s rank correlation coefficients based on energy-adjusted values (other than energy intake and total fat in % energy) and expressed as deattenuated CC. *P < 0.05 where *r* ≥ 0.20, **P < 0.01 where *r* ≥ 0.26 for Long-FFQ, *P < 0.05 where *r* ≥ 0.21, and **P < 0.01 where *r* ≥ 0.27 for short-FFQ among men.

^c^*Deattenuated CCx = observed CCx × SQRT(1 + λx/n)*, where λx is the ratio of within- to between-individual variance for nutrient x, and n is number of dietary records.

**Table 3.** Energy and nutrient intakes according to long-FFQ/short-FFQ, percentage differences between intakes by two FFQs and 12d-WFR and their correlations in women

	Women											

	Long-FFQ (*n* = 142)						Short-FFQ (*n* = 136)					
	
	12d-WFR		FFQ		%^a^	CC^b,c^	12d-WFR		FFQ		%^a^	CC^b,c^
			
	Mean	(SD)	Mean	(SD)			Mean	(SD)	Mean	(SD)		
Energy, kcal	1805	(309)	2036	(671)	13**	0.17^c^	1810	(309)	1382	(384)	−24**	0.16^c^
Water, g	2321	(551)	2665	(1009)	15**	0.48**	2335	(536)	1891	(743)	−19**	0.58**
Protein, g	70.0	(14.8)	76.7	(30.0)	10*	0.33**	70.2	(14.8)	53.4	(18.8)	−24**	0.43**
Sum of amino acid residues	24.0	(5.9)	31.6	(13.3)	31**	0.37**	24.1	(5.8)	23.5	(8.4)	−3	0.43**
Total fat, g	54.6	(14.1)	64.5	(29.8)	18**	0.33**	54.7	(14.4)	38.0	(17.8)	−30**	0.29**

Total fat in % energy	27.0	(4.0)	27.5	(5.7)	2	0.34^c^**	27.0	(3.9)	23.9	(6.0)	−11**	0.14^c^
Saturated fatty acid, g	15.2	(4.9)	19.0	(10.5)	26**	0.46**	15.2	(5.0)	10.6	(5.7)	−30**	0.44**
Monounsaturated fatty acid, g	19.2	(5.1)	23.6	(11.1)	23**	0.21*	19.2	(5.2)	14.0	(7.0)	−27**	0.21*
Polyunsaturated fatty acid, g	11.7	(3.0)	14.1	(6.4)	20**	0.28**	11.8	(3.0)	8.6	(3.8)	−27**	0.24*
n-3 PUFA	2.4	(0.8)	2.7	(1.4)	16**	0.40**	2.4	(0.8)	1.9	(1.0)	−20**	0.30**
n-6 PUFA	9.2	(2.4)	11.3	(5.2)	23**	0.28**	9.2	(2.4)	6.7	(2.9)	−28**	0.24*
Triacylglycerol equivalents, g	47.0	(12.3)	59.3	(27.6)	26**	0.33**	47.2	(12.5)	34.6	(16.2)	−27**	0.31**
Cholesterol, mg	304.4	(89.4)	316.5	(229.4)	4	0.38**	303.8	(88.6)	214.8	(109.7)	−29**	0.46**

Carbohydrate, g	248.4	(40.5)	276.7	(87.0)	11**	0.40**	248.9	(39.5)	194.3	(50.3)	−22**	0.44**

Total dietary fiber, g	16.5	(5.2)	17.6	(8.6)	7	0.61**	16.6	(5.1)	10.7	(5.0)	−36**	0.57**
Water soluble fiber, g	3.7	(1.4)	4.2	(2.1)	12**	0.60**	3.8	(1.4)	2.4	(1.3)	−35**	0.54**
Water insoluble fiber, g	12.1	(3.7)	12.9	(6.2)	6	0.60**	12.2	(3.7)	7.9	(3.6)	−35**	0.59**

Sodium, mg	3809	(921)	4480	(2080)	18**	0.38**	3805	(906)	2876	(1295)	−24**	0.39**
Potassium, mg	2968	(800)	3610	(1587)	22**	0.54**	2992	(778)	2300	(935)	−23**	0.47**
Calcium, mg	590	(205)	754	(474)	28**	0.42**	593	(206)	365	(190)	−38**	0.60**
Magnesium, mg	294	(78)	359	(143)	22**	0.51**	296	(77)	255	(93)	−14**	0.45**
Phosphorus, mg	1092	(249)	1278	(537)	17**	0.37**	1097	(248)	810	(281)	−26**	0.54**
Iron, mg	8.8	(2.6)	9.7	(3.8)	10**	0.57**	8.9	(2.6)	7.2	(2.7)	−19**	0.63**
Zinc, mg	7.9	(1.6)	8.7	(3.1)	11**	0.27**	7.9	(1.6)	6.1	(1.9)	−23**	0.38**
Copper, mg	1.25	(0.30)	1.38	(0.49)	10**	0.49**	1.26	(0.29)	0.95	(0.30)	−25**	0.65**
Manganese, mg	4.16	(1.44)	4.31	(1.77)	4	0.74**	4.19	(1.42)	3.23	(1.25)	−23**	0.68**
Iodine, µg	1829	(4031)	252	(251)	−86**	0.14	1686	(3390)	183	(185)	−89**	0.15
Selenium, µg	48	(12)	64	(30)	35**	0.15	47	(12)	45	(22)	−6	0.30**
Chromium, µg	7	(2)	8	(4)	13*	0.26**	7	(2)	4	(2)	−44**	0.26**
Molybdenum, µg	172	(52)	224	(96)	31**	0.57**	173	(52)	195	(61)	13**	0.69**

Retinol, µg	203	(179)	343	(474)	69**	0.30**	204	(181)	272	(370)	33*	0.34**
Alpha-carotene, µg	441	(258)	672	(1030)	52**	0.56**	450	(260)	610	(503)	36**	0.37**
Beta-carotene, µg	3665	(1542)	4443	(4016)	21**	0.50**	3721	(1539)	3647	(2548)	−2	0.37**
Cryptoxanthin, µg	441	(347)	1335	(1580)	203**	0.37**	446	(352)	1262	(1540)	183**	0.40**
Beta carotene equivalents, µg	4279	(1759)	5446	(4698)	27**	0.51**	4348	(1752)	4565	(3017)	5	0.34**
Retinol equivalents, µg	575	(263)	800	(668)	39**	0.41**	583	(263)	655	(471)	12	0.34**
Vitamin D, µg	9.0	(5.1)	9.6	(6.9)	7	0.49**	9.1	(5.1)	8.1	(5.3)	−10	0.47**
Alpha-tocopherol, mg	8.0	(2.7)	9.3	(4.6)	16**	0.50**	8.1	(2.7)	5.6	(2.9)	−32**	0.49**
Beta-tocopherol, mg	0.3	(0.1)	0.4	(0.2)	26**	0.24*	0.3	(0.1)	0.3	(0.1)	−22**	0.36**
Gamma-tocopherol, mg	10.1	(2.9)	12.3	(6.4)	21**	0.29**	10.2	(2.9)	7.4	(4.1)	−27**	0.29**
Delta-tocopherol, mg	2.6	(0.9)	2.8	(1.6)	6	0.53**	2.7	(0.9)	2.1	(1.2)	−20**	0.53**
Vitamin K, µg	294	(113)	341	(236)	16**	0.52**	297	(113)	251	(155)	−15**	0.53**
Vitamin B_1_, mg	1.02	(0.36)	1.10	(0.45)	8	0.36**	1.03	(0.37)	0.76	(0.31)	−26**	0.37**
Vitamin B_2_, mg	1.49	(0.44)	1.65	(0.80)	10*	0.43**	1.50	(0.44)	1.08	(0.46)	−28**	0.61**
Niacin, mg	18.4	(5.4)	22.6	(8.8)	23**	0.32**	18.5	(5.4)	18.2	(7.0)	−2	0.18
Vitamin B_6_, mg	1.45	(0.57)	1.61	(0.67)	11**	0.59**	1.46	(0.58)	1.19	(0.45)	−19**	0.46**
Vitamin B_12_, µg	7.3	(3.5)	7.5	(4.6)	3	0.35**	7.3	(3.5)	6.6	(4.0)	−11*	0.46**
Folate, µg	445	(147)	484	(239)	9*	0.62**	449	(145)	313	(154)	−30**	0.55**
Pantothenic acid, mg	6.36	(1.57)	8.08	(3.48)	27**	0.46**	6.41	(1.56)	5.30	(1.90)	−17**	0.61**
Biotin, µg	31.4	(8.7)	44.0	(18.1)	40**	0.36**	31.7	(8.5)	34.4	(13.5)	9*	0.32**
Vitamin C, mg	155	(72)	184	(110)	18**	0.66**	156	(72)	94	(63)	−40**	0.59**

Daidzein, mg	13.17	(7.31)	17.28	(12.43)	31**	0.55**	13.33	(7.40)	15.61	(11.12)	17**	0.64**
Genistein, mg	22.34	(12.53)	28.57	(20.88)	28**	0.53**	22.61	(12.68)	25.52	(19.04)	13*	0.63**

Ethanol, g	4.6	(9.6)	4.3	(8.9)	−7	0.67**	4.6	(9.7)	NA	NA	NA	NA

**MEDIAN**						**0.43**						**0.44**

12d-WFR, 12-day weighed food records; CC, correlation coefficient; FFQ, Food Frequency Questionnaire; PUFA, poly-unsaturated fatty acids; NA, not applicable for calculation; SD, standard deviation.

^a^Percentage differences: (FFQ − 12d-WFR)/12d-WFR × 100 (%). *P*-values refer to paired *t*-test between intakes by each FFQs and those by 12d-WFR for each; *P < 0.05, **P < 0.01.

^b^Spearman’s rank correlation coefficients based on energy-adjusted values (other than energy intake and total fat in % energy) and expressed as deattenuated CC. For women, *P < 0.05 where *r* ≥ 0.20, and **P < 0.01 where *r* ≥ 0.26.

^c^*Deattenuated CCx = observed CCx × SQRT(1 + λx/n)*, where λx is the ratio of within- to between-individual variance for nutrient x, and n is number of dietary records.

Table 4 shows the daily intake of 29 food groups and the number of food items (and supplemental questions) listed in the long- and short-FFQs. Most food groups in the short-FFQ had fewer items than in the long-FFQ. Accordingly, intake of most food groups based on the short-FFQ tended to be more underestimated than those based on the long-FFQ among men and women. Further, items related to potato and starches, sugar, and processed meat were not listed in the short-FFQ, so intake of these food groups could not be estimated for the short-FFQ. The median (range) values across deattenuated CCs of food group intakes based on the long- and short-FFQs were 0.46 (0.22–0.75) and 0.46 (0.16–0.68) in men and 0.48 (0.06–0.80) and 0.44 (−0.21 to 0.78) in women.

**Table 4.** Food-group intakes according to long-FFQ/short-FFQ, percentage differences between intakes by two FFQs and 12d-WFR and their correlations in men and women

	*n* of items (supplemental questions)^d^		Men								Women							
	
			Long-FFQ (*n* = 98)				Short-FFQ (*n* = 92)				Long-FFQ (*n* = 142)				Short-FFQ (*n* = 136)			
			
			12d-WFR	FFQ	%^a^	CC^b,c^	12d-WFR	FFQ	%^a^	CC^b,c^	12d-WFR	FFQ	%^a^	CC^b,c^	12d-WFR	FFQ	%^a^	CC^b,c^
							
	Long- FFQ	Short- FFQ	Mean (SD)	Mean (SD)			Mean (SD)	Mean (SD)			Mean (SD)	Mean (SD)			Mean (SD)	Mean (SD)		
			g	g			g	g			g	g			g	g		
Cereals	9 (4)	4	508 (138)	637 (264)	25	0.60**	508 (140)	538 (199)	6	0.57**	348 (80)	472 (131)	35	0.31**	348 (81)	403 (101)	16	0.45**
Rice	1 (3)	1 (3)	394 (144)	460 (177)	17	0.58**	394 (145)	449 (185)	14	0.57**	259 (86)	326 (100)	26	0.48**	261 (86)	325 (100)	24	0.57**
Potatoes and starches	5	0	45 (29)	37 (29)	−17	0.28**	45 (29)	NANA	NA	NA	43 (25)	44 (31)	2	0.45**	44 (25)	NANA	NA	NA
Sugar	1 (2)	0	6 (3)	0 (2)	−91	0.34**	6 (3)	NANA	NA	NA	6 (3)	1 (1)	−91	0.23*	6 (3)	NANA	NA	NA
Pulses	9	4	70 (47)	78 (105)	11	0.62**	72 (48)	69 (102)	−4	0.63**	70 (44)	77 (66)	10	0.61**	71 (45)	64 (76)	−10	0.59**
Vegetables	38	14	358 (163)	277 (217)	−23	0.59**	365 (162)	138 (106)	−62	0.49**	344 (133)	359 (227)	4	0.59**	350 (131)	188 (125)	−46	0.42**
Green and yellow	19	8	123 (67)	132 (153)	7	0.52**	126 (67)	78 (72)	−38	0.53**	127 (63)	156 (114)	23	0.59**	129 (62)	104 (76)	−19	0.44**
White vegetables	19	7	234 (119)	145 (104)	−38	0.51**	239 (119)	60 (51)	−75	0.33**	218 (95)	202 (132)	−7	0.50**	221 (94)	84 (65)	−62	0.23*
Pickled vegetables	7	6	14 (13)	33 (49)	140	0.43**	14 (13)	23 (30)	62	0.37**	15 (16)	46 (54)	213	0.54**	15 (17)	31 (37)	108	0.51**
Cruciferous vegetables	7	2	118 (71)	55 (50)	−53	0.46**	119 (71)	16 (18)	−87	0.41**	118 (63)	81 (66)	−32	0.43**	120 (64)	24 (22)	−80	0.26**
Fruits	21	6	94 (79)	147 (127)	56	0.75**	96 (80)	98 (116)	2	0.60**	139 (85)	260 (230)	88	0.56**	139 (85)	150 (134)	7	0.50**
Citrus fruit	4	2	22 (30)	43 (42)	99	0.43**	21 (31)	46 (63)	115	0.46**	36 (32)	103 (159)	188	0.39**	36 (32)	80 (99)	124	0.42**
Other fruit	16	3	72 (66)	102 (102)	41	0.74**	75 (66)	50 (76)	−33	0.68**	103 (69)	155 (126)	51	0.56**	104 (69)	68 (52)	−35	0.54**
Fungi	3	1	21 (16)	11 (11)	−49	0.26**	21 (16)	5 (11)	−78	0.29**	18 (12)	16 (12)	−12	0.41**	18 (12)	6 (8)	−68	0.27**
Algae	3	2	10 (11)	8 (10)	−22	0.25*	10 (11)	7 (11)	−27	0.22*	9 (10)	9 (15)	0	0.32**	9 (10)	8 (8)	−13	0.26**
Fish and shellfish	21	11	108 (44)	76 (48)	−30	0.30**	109 (44)	60 (41)	−44	0.30**	82 (35)	76 (53)	−6	0.47**	82 (35)	60 (39)	−26	0.56**
Meats	19	12	87 (36)	71 (48)	−19	0.44**	89 (36)	56 (44)	−37	0.38**	61 (27)	56 (38)	−8	0.29**	61 (27)	43 (32)	−30	0.44**
Processed meat	4	0	17 (12)	10 (10)	−40	0.34**	18 (12)	NANA	NA	NA	12 (8)	9 (8)	−20	0.60**	11 (8)	NANA	NA	NA
Red meat	9	9	46 (23)	40 (31)	−13	0.43**	47 (23)	46 (39)	−2	0.40**	32 (18)	30 (23)	−6	0.20*	32 (18)	36 (28)	14	0.34**
Poultry	4	1	21 (16)	19 (20)	−10	0.22*	22 (16)	7 (7)	−68	0.30**	17 (11)	16 (16)	−4	0.56**	17 (11)	5 (6)	−69	0.29**
Eggs	1	1	40 (18)	44 (73)	10	0.56**	40 (18)	44 (67)	8	0.52**	32 (14)	37 (49)	14	0.48**	32 (14)	27 (17)	−16	0.50**
Milk and dairy products	6 (2)	2	105 (84)	200 (263)	90	0.58**	108 (84)	100 (161)	−7	0.59**	147 (101)	314 (340)	114	0.52**	148 (102)	113 (120)	−23	0.73**
Fats and oils	2 (7)	0 (7)	12 (5)	12 (6)	1	0.30**	12 (5)	8 (5)	−35	0.16	10 (4)	14 (8)	44	0.06	10 (4)	8 (4)	−17	−0.21
Confectionaries	6	1	31 (27)	18 (23)	−42	0.61**	32 (28)	8 (11)	−75	0.50**	46 (32)	28 (23)	−39	0.48**	46 (32)	12 (16)	−74	0.26**
Alcoholic beverages	6	0	350 (313)	420 (384)	20	0.72**	360 (319)	NANA	NA	NA	82 (166)	88 (198)	8	0.66**	82 (168)	NANA	NA	NA
Non-alcoholic beverages	10	6	600 (385)	709 (430)	18	0.34**	608 (388)	534 (377)	−12	0.34**	673 (381)	694 (417)	3	0.46**	670 (379)	591 (318)	−12	0.46**
Green tea	4	2	314 (335)	337 (373)	7	0.66**	324 (342)	292 (367)	−10	0.64**	392 (331)	401 (419)	2	0.80**	393 (328)	355 (310)	−10	0.78**
Coffee	3	3	123 (148)	299 (261)	143	0.51**	125 (149)	242 (196)	94	0.63**	119 (178)	236 (204)	98	0.56**	121 (181)	236 (197)	94	0.62**
Seasonings and spices	8 (1)	1 (1)	138 (74)	22 (12)	−84	0.25*	137 (73)	16 (11)	−88	0.41**	114 (80)	23 (13)	−80	0.16	113 (77)	15 (10)	−87	0.16

**MEDIAN**						**0.46**				**0.46**				**0.48**				**0.44**

12d-WFR, 12-day weighed food records; CC, correlation coefficient; FFQ, Food Frequency Questionnaire; NA, not applicable for calculation; SD, standard deviation.

^a^Percentage differences: (FFQ − 12d-WFR)/12d-WFR × 100 (%).

^b^Spearman’s rank correlation coefficients based on energy-adjusted values (other than energy intake and total fat in % energy) and expressed as deattenuated CC. *P < 0.05 where *r* ≥ 0.20, and **P < 0.01 where *r* ≥ 0.26 for Long-FFQ, *P < 0.05 where *r* ≥ 0.21, and **P < 0.01 where *r* ≥ 0.27 for short-FFQ among men, For women, *P < 0.05 where *r* ≥ 0.20, and **P < 0.01 where *r* ≥ 0.26.

^c^*Deattenuated CCx = observed CCx × SQRT(1 + λx/n)*, where λx is the ratio of within- to between-individual variance for nutrient x, and n is number of dietary records.

^d^Supplemental questions were supporting for some kinds of the foods, and were not included in the number of items. 2 items of nuts and seeds and 2 items of drink water were not shown, because a few number of food groups, respectively.

In area-adjusted analysis, the median values of CCs for the either FFQs did not differ substantially (data not shown).

### Cross classification by quintile

Table 5, Table 6, and Table 7 show the results of comparison of the long- and short-FFQs with the 12d-WFR and of the short-FFQ with the long-FFQ for energy-adjusted nutrients and food groups, based on cross-classification by quintile in men and women. Comparing classification by the short-FFQ and 12d-WFR, the proportion of subjects classified into the opposite extreme category was 5% or less for most nutrients in men and women, as well as for many food groups. Regarding the agreement of classification by the two FFQs, the proportion of subjects classified into the opposite extreme category was 3% or less for almost all nutrients and food groups in men and women. In addition, the proportion of subjects classified into the same or adjacent category was more than 75% for many nutrients and for approximately half of the food groups, with median values of 80% and 76% in men and women, respectively, for nutrients, and 76% and 74% in men and women, respectively, for food groups.

**Table 5.** Comparison of long- and short-FFQs with 12d-WFR and short-FFQ with long-FFQ for energy-adjusted nutrients, based on cross-classification by quintile (%) in men

	Men								

	long-FFQ vs 12d-WFR (*n* = 98)			short-FFQ vs 12d-WFR (*n* = 92)			short-FFQ vs long-FFQ (*n* = 92)		
		
	Same category	Same and adjacent category	Extreme category	Same category	Same and adjacent category	Extreme category	Same category	Same and adjacent category	Extreme category
Energy^a^	27	67	2	27	68	1	39	76	0
Water	26	70	6	36	62	1	35	75	1
Protein	29	61	0	24	62	0	43	83	1
Sum of amino acid residues	24	67	3	17	59	4	39	71	2
Total fat	34	72	4	33	73	1	42	80	0

Total fat in % energy^a^	32	68	4	35	73	3	36	74	1
Saturated fatty acid	30	67	1	26	66	3	32	70	1
Monounsaturated fatty acid	32	74	1	25	66	3	32	73	1
Polyunsaturated fatty acid	28	61	2	29	65	2	34	77	0
n-3 PUFA	21	60	2	29	67	4	51	84	0
n-6 PUFA	28	62	1	33	64	1	38	76	0
Triacylglycerol equivalents	29	71	4	24	66	2	37	78	0
Cholesterol	33	76	2	28	71	2	47	82	0

Carbohydrate	45	82	0	49	76	2	49	91	0

Total dietary fiber	43	84	2	37	78	0	48	77	0
Water soluble fiber	43	77	2	35	78	1	50	80	1
Water insoluble fiber	51	83	1	32	75	0	46	80	0

Sodium	27	60	2	27	63	0	48	88	1
Potassium	32	69	5	29	72	3	40	78	0
Calcium	33	72	0	38	74	1	38	82	1
Magnesium	33	65	4	33	64	5	40	82	0
Phosphorus	35	65	1	27	70	2	39	80	1
Iron	37	73	2	29	73	0	48	84	0
Zinc	36	67	3	26	66	3	48	80	0
Copper	45	81	3	32	74	2	51	90	1
Manganese	41	72	0	35	65	0	39	84	1
Iodine	22	57	9	27	59	5	41	72	1
Selenium	23	51	4	26	52	4	43	79	3
Chromium	26	67	2	33	64	1	46	83	1
Molybdenum	33	76	1	36	70	3	38	87	1

Retinol	21	63	3	26	58	2	32	70	7
Alpha-carotene	24	63	4	27	70	3	36	77	0
Beta-carotene	27	69	1	30	73	3	38	78	2
Cryptoxanthin	40	77	1	30	64	2	34	70	0
Beta carotene equivalents	26	71	2	33	67	4	38	76	2
Retinol equivalents	29	64	3	23	63	4	37	68	2
Vitamin D	22	62	4	21	62	1	41	84	2
Alpha-tocopherol	37	73	4	30	67	3	42	85	1
Beta-tocopherol	29	65	1	28	64	1	42	84	0
Gamma-tocopherol	21	55	2	28	64	4	39	76	1
Delta-tocopherol	30	69	3	24	73	3	47	84	0
Vitamin K	32	71	1	35	77	0	51	88	0
Vitamin B_1_	30	65	3	30	61	4	34	75	0
Vitamin B_2_	33	66	2	39	71	2	42	80	0
Niacin	26	63	5	15	60	2	35	77	0
Vitamin B_6_	26	72	3	32	66	2	42	78	0
Vitamin B_12_	22	62	1	26	64	4	47	84	0
Folate	36	80	1	34	71	0	43	82	0
Pantothenic acid	33	77	1	38	78	2	53	87	0
Biotin	26	60	6	30	62	4	36	73	1
Vitamin C	38	74	0	27	71	0	43	80	0

Daidzein	40	72	0	35	77	1	48	84	0
Genistein	42	74	0	33	76	1	46	83	0

Ethanol	52	88	1	NA	NA	NA	NA	NA	NA

**MEDIAN**	**31**	**69**	**2**	**30**	**67**	**2**	**41**	**80**	**0**

12d-WFR, 12-day weighed food record; FFQ, Food Frequency Questionnaire; NA, not applicable for calculation; PUFA, poly-unsaturated fatty acids.

^a^Cross-classifications for energy intake and total fat in % energy were calculated by using crude values.

**Table 6.** Comparison of long- and short-FFQs with 12d-WFR and short-FFQ with long-FFQ for energy-adjusted nutrients, based on cross-classification by quintile (%) in women

	Women								

	long-FFQ vs 12d-WFR (*n* = 142)			short-FFQ vs 12d-WFR (*n* = 136)			short-FFQ vs long-FFQ (*n* = 136)		
		
	Same category	Same and adjacent category	Extreme category	Same category	Same and adjacent category	Extreme category	Same category	Same and adjacent category	Extreme category
Energy^a^	27	56	6	26	57	4	42	77	2
Water	30	74	3	37	75	1	35	82	1
Protein	28	61	4	31	66	1	32	72	4
Sum of amino acid residues	30	66	3	33	69	4	32	72	2
Total fat	30	67	1	26	63	2	29	66	4

Total fat in % energy^a^	29	64	4	23	54	5	26	64	3
Saturated fatty acid	28	73	2	26	69	2	31	63	1
Monounsaturated fatty acid	30	63	6	25	62	4	24	58	4
Polyunsaturated fatty acid	28	58	3	24	62	4	35	69	4
n-3 PUFA	23	63	2	26	60	4	34	68	1
n-6 PUFA	30	60	4	26	64	6	36	71	4
Triacylglycerol equivalents	25	63	2	32	65	4	26	65	4
Cholesterol	28	65	4	29	68	2	38	78	3

Carbohydrate	24	65	3	32	65	1	31	68	1

Total dietary fiber	33	77	1	34	77	2	38	79	2
Water soluble fiber	40	77	1	34	72	3	40	76	1
Water insoluble fiber	34	74	1	36	76	1	37	79	2

Sodium	28	65	2	26	68	3	35	76	1
Potassium	33	70	1	27	69	2	40	79	1
Calcium	29	73	4	42	75	1	35	70	3
Magnesium	35	71	2	32	68	2	43	79	3
Phosphorus	29	64	2	35	74	2	35	72	4
Iron	40	76	3	42	76	1	40	82	1
Zinc	25	68	5	33	67	1	34	71	7
Copper	35	73	1	35	79	1	46	84	1
Manganese	44	85	0	41	79	1	43	82	1
Iodine	24	54	5	22	60	9	40	74	2
Selenium	22	57	5	18	60	4	41	67	2
Chromium	25	60	4	30	60	5	33	64	4
Molybdenum	33	70	1	40	79	1	46	79	1

Retinol	31	59	5	24	63	4	33	70	4
Alpha-carotene	28	66	1	32	68	4	39	76	0
Beta-carotene	32	70	1	25	60	2	43	78	2
Cryptoxanthin	25	63	4	24	68	4	41	79	2
Beta carotene equivalents	38	70	1	24	62	1	38	76	2
Retinol equivalents	33	64	1	30	62	4	35	70	2
Vitamin D	30	70	3	32	67	2	39	76	1
Alpha-tocopherol	35	65	2	32	69	1	36	78	1
Beta-tocopherol	20	57	4	28	59	4	35	76	3
Gamma-tocopherol	27	62	2	24	63	5	32	77	1
Delta-tocopherol	35	68	2	29	70	1	35	75	1
Vitamin K	32	66	1	35	70	1	35	76	0
Vitamin B_1_	30	58	1	33	67	4	40	70	2
Vitamin B_2_	35	65	4	40	75	1	39	77	4
Niacin	27	58	3	24	59	8	41	79	1
Vitamin B_6_	34	74	1	34	70	4	40	76	1
Vitamin B_12_	28	61	4	29	65	2	35	79	1
Folate	38	73	1	32	74	1	35	78	1
Pantothenic acid	30	71	1	30	76	1	32	69	2
Biotin	31	61	4	28	60	2	43	79	1
Vitamin C	38	77	1	33	75	1	41	80	2

Daidzein	32	71	2	40	76	1	41	81	1
Genistein	32	68	1	38	76	1	45	85	1

Ethanol	46	77	0	NA	NA	NA	NA	NA	NA

**MEDIAN**	**30**	**66**	**2**	**31**	**68**	**2**	**36**	**76**	**2**

12d-WFR, 12-day weighed food record; FFQ, Food Frequency Questionnaire; NA, not applicable for calculation; PUFA, poly-unsaturated fatty acids.

^a^Cross-classifications for energy intake and total fat in % energy were calculated by using crude values.

**Table 7.** Comparison of long- and short-FFQs with 12d-WFR and short-FFQ with long-FFQ for energy-adjusted food groups, based on cross-classification by quintile (%) in men and women

	Men								

	Long-FFQ vs 12d-WFR (*n* = 98)			Short-FFQ vs 12d-WFR (*n* = 92)			Short-FFQ vs Long-FFQ (*n* = 92)		
		
	Same category	Same and adjacent category	Extreme category	Same category	Same and adjacent category	Extreme category	Same category	Same and adjacent category	Extreme category
Cereals	39	69	1	41	74	2	50	82	1
Rice	41	73	1	36	76	2	43	77	1
Potatoes and starches	27	60	5	NA	NA	NA	NA	NA	NA
Sugar	27	64	2	NA	NA	NA	NA	NA	NA
Pulses	38	72	1	35	75	3	37	80	1
Vegetables	36	78	0	27	71	3	38	85	1

Green and yellow	30	73	3	25	71	2	36	76	1
White vegetables	31	69	1	26	61	2	45	76	2
Pickled vegetables	30	64	2	25	63	5	47	87	0
Cruciferous vegetables	34	69	3	34	70	5	39	73	2

Fruits	38	82	0	33	71	2	35	76	1
Citrus fruit	30	70	2	27	66	1	36	70	0
Other fruit	38	81	0	36	76	1	33	75	0
Fungi	18	59	5	32	59	4	26	71	3
Algae	21	56	5	27	51	5	32	73	1
Fish and shellfish	23	59	1	39	64	7	32	79	2

Meats	34	67	5	27	65	2	37	70	0
Processed meat	20	60	3	NA	NA	NA	NA	NA	NA
Red meat	31	67	4	25	65	4	38	73	1
Poultry	21	58	5	21	59	2	35	71	4
Eggs	30	76	4	28	68	2	45	82	0
Milk and dairy products	34	77	2	33	79	0	34	74	1
Fats and oils	31	64	3	23	55	7	35	67	2
Confectionaries	33	76	1	22	75	1	30	79	1
Alcoholic beverages	45	83	1	NA	NA	NA	NA	NA	NA
Non-alcoholic beverages	33	67	6	24	65	5	37	74	3
Green tea	33	78	0	34	77	0	48	87	1
Coffee	26	67	1	33	70	0	55	88	0
Seasonings and spices	20	60	5	28	71	4	48	87	0

**MEDIAN**	**31**	**69**	**2**	**28**	**70**	**2**	**37**	**76**	**1**

	Women								

	long-FFQ vs 12d-WFR (*n* = 142)			short-FFQ vs 12d-WFR (*n* = 136)			short-FFQ vs long-FFQ (*n* = 136)		

Cereals	25	61	4	34	69	1	28	69	4
Rice	33	76	5	33	72	1	43	82	2
Potatoes and starches	27	64	5	NA	NA	NA	NA	NA	NA
Sugar	30	58	5	NA	NA	NA	NA	NA	NA
Pulses	34	74	1	35	71	3	43	83	1
Vegetables	30	76	1	29	68	3	39	75	4

Green and yellow	32	73	1	31	62	1	42	78	1
White vegetables	32	67	2	23	60	4	39	70	4
Pickled vegetables	28	71	4	29	72	1	41	88	2
Cruciferous vegetables	32	67	5	30	63	4	29	68	1

Fruits	31	73	3	32	67	1	34	78	0
Citrus fruit	29	68	4	32	63	2	40	79	1
Other fruit	36	72	1	26	74	1	36	70	1
Fungi	29	65	4	21	55	3	25	65	4
Algae	20	62	5	25	63	7	32	74	4
Fish and shellfish	27	66	3	26	68	1	34	71	1

Meats	27	54	5	28	61	4	36	67	3
Processed meat	34	68	3	NA	NA	NA	NA	NA	NA
Red meat	26	59	7	27	58	4	35	70	4
Poultry	30	64	4	23	56	7	26	62	5
Eggs	29	66	4	26	64	2	43	79	1
Milk and dairy products	31	74	2	40	85	2	29	67	3
Fats and oils	25	52	8	15	47	10	24	65	6
Confectionaries	30	71	2	22	60	6	29	65	4
Alcoholic beverages	44	78	0	NA	NA	NA	NA	NA	NA
Non-alcoholic beverages	30	70	3	32	67	1	38	80	1
Green tea	46	87	0	46	79	0	40	86	0
Coffee	31	69	1	32	75	1	65	95	0
Seasonings and spices	19	57	6	23	56	4	36	78	1

**MEDIAN**	**30**	**68**	**4**	**29**	**64**	**2**	**36**	**74**	**2**

12d-WFR, 12-day weighed food record; FFQ, Food Frequency Questionnaire; NA, not applicable for calculation.

## DISCUSSION

We developed a short version of the long-FFQ used in the baseline survey of the JPHC-NEXT protocol and compared the validity of the intake estimates obtained with the two versions for middle-aged and elderly subjects in five cohort regions specified in the study protocol. The deattenuated energy-adjusted CCs between the intake based on both the long- and short-FFQs and the 12d-WFR were moderate or high for the intake of many nutrients and food groups. These CCs of short-FFQ (vs the 12d-WFR) for the nutrient and food groups were similar to those for the long-FFQ, and the classification of subjects according to quintile by the two FFQs was concordant.

Correlations between the intakes based on the long-FFQ and 12d-WFR were moderate or high for many nutrients and food groups for both men and women, although they were very low for some nutrients, such as iodine. The median CCs between the long-FFQ and the 12d-WFR were around 0.40 or 0.50 across nutrients and food groups. For many nutrients and food groups, these CCs were similar to or slightly better than those for the JPHC-FFQ^9^^,^^10^ (median values: 0.29 to 0.41). Moreover, comparison with the median CCs (vs 1- to 63-day food records) obtained across nutrients (0.31 to 0.56) in 21 validation studies of FFQs developed in Japan^3^ showed no inferiority in the validity of our long-FFQ for many nutrients and food groups. In studies on the validity of FFQs (usually assessing over 100 food items) in other countries, the CCs of energy-adjusted nutrients have ranged from 0.45 to 0.70.^22^ Compared with these, our FFQs showed similar performance for many nutrients and food groups in ranking individuals according to the estimates. Thus, the accuracy of the estimates obtained with our long-FFQ is comparable to that of estimates obtained with previously developed long versions of FFQs. The low CC for estimated iodine intake may be attributable to the fact that it could not be considered in development of the food list for either the original JPHC-FFQ^8^ or in the modification of the long-FFQ because iodine was an additional nutrient item for the revised food composition table^19^ used for this calculation. The long-FFQ provided reasonably valid measures of consumption for many nutrients and food groups in middle-aged and elderly Japanese.

However, all of the nutrient and food group estimates based on the long-FFQ tended to be overestimates, particularly for women, and the CC for estimated energy intake was much lower than in previous studies. The small contribution of individual foods to total energy intake was likely attributable to errors in responses for foods on the predetermined list. The percentage contribution of the top 20 foods to energy intake was 77% in the United States between 2003 and 2006^23^ and 64% for men and 56% for women in Japan in 1994.^24^ In this study, the percentage contribution of these foods (based on the 12d-WFR) was 53% for men and 45% for women, indicating that the relatively low validity of estimates of energy intake from FFQs, especially among women, may be explained by differences in dietary variety between countries, time periods, or genders.^1^^,^^9^ These findings suggest that understanding the absolute values for intake is difficult even with the present 172-item long-FFQ, especially in ranking correlations of total energy intake for women. Regarding cross-classification of energy-adjusted intakes based on each FFQ with the 12d-WFR, many nutrient and food group results of these FFQs can be used to rank individuals.

Compared with the 12d-WFR and the long-FFQ, the intake of nearly all nutrients and food groups of the short-FFQ were underestimated for men and women. Accordingly, absolute intake values cannot be ascertained from the short-FFQ, and the intake estimates from the short-FFQ cannot be compared directly with those of the long-FFQ. Further, intakes of some nutrients were higher in both the short- and long-FFQs than those based on the 12d-WFR. These nutrients might be influenced by contributions of both absolute intake and intra-individual variation from specific foods. For example, based on the original JPHC-FFQ, ≥55% of total retinol intake was derived from only two items (“pork liver” and “chicken liver”) in the long-FFQ, ≥80% of cryptoxanthin was derived from one item (“mandarin orange”), and ≥60% of isoflavone was derived from three items (“fermented soybeans”, “*tofu* [bean curd]” and “boiled bean curd”) (data not shown). These items were also included in the short- and long-FFQs, so intakes of such nutrients might be equally estimated with both FFQs. However, correlations with intakes based on the 12d-WFR were moderate or high for many nutrients and food groups, and good concordance was seen with classification according to quintile of intake based on the 12d-WFR. Of the FFQ validation studies that have been conducted in Japan, 14 studies of short-FFQs with 70 items or fewer reported median correlations for nutrients ranging from 0.31 to 0.45.^3^ Compared with these FFQs, the short-FFQ we developed provides more accurate estimates for many nutrients and food groups.

Moreover, median CCs for short-FFQs are not necessarily inferior to those for long-FFQs. Several validation studies of long and short versions of FFQs^5^^,^^6^^,^^25^^,^^26^ have reported CCs of long and short versions of FFQs around 0.50 and around 0.30 to 0.50, respectively. The median intake estimates obtained with our present short-FFQ were comparable to or higher than those in these previous studies, suggesting that the differences in estimate accuracy between the long and short versions were within an acceptable range for many nutrients.

Moreover, characteristics of CCs for each nutrient and food group based on the short-FFQ compared with the 12d-WFR were closely similar to those for the long-FFQ among men and women. This is likely due to the fact that, in developing the short-FFQ, the foods included, which were based on the long-FFQ food list, were items that contribute heavily to inter-individual variation in the intake of each nutrient. In addition, classification of the intake estimates obtained with both FFQs according to quintile showed high concordance between these two different FFQs for many nutrients and food groups. Therefore, the short-FFQ shows promise for use in updating intake rankings obtained using long-FFQs in cohort studies that require follow-up.

Our study has several limitations. First, subjects were not selected by random sampling. Maintaining a weighed food record requires a high level of motivation, which may have resulted in a greater proportion of highly health-conscious individuals than exist in the general population.^1^ Although the characteristics of the subjects indicated that they were not always as highly motivated and health conscious as participants in the 2012 National Health and Nutrition Survey in Japan,^27^ the possibility that our results were overestimates cannot be ruled out. Second, certain food groups in the short-FFQ do not include any food items. This is because we developed the short-FFQ by selecting foods that contribute to inter-individual variation in nutrient intake, so those that do not contribute to nutrition were not included in the list. Calculations cannot be performed for these food groups using the short-FFQ, so their intakes cannot be estimated. In contrast, Strengths of the study include its simultaneous examination of the validity of ranking individuals for nutrient and food group estimates by the short- and long-FFQs among residents of the study area.

In conclusion, our findings indicate that both short- and long-FFQs of the JPHC-NEXT cohort provide similar levels of ranking accuracy for the same nutrients and food groups, and that intakes can be comparably ranked regardless of the number of items in the FFQ. In addition, the correlations between the intake estimates obtained with both FFQs and those obtained with the 12d-WFR showed moderate validity for many nutrients and food groups. This study suggests that the short-FFQ is reasonable for use in the follow-up of baseline surveys with the long-FFQ.

## ONLINE ONLY MATERIAL

Abstract in Japanese.
