# Supplementary material for: Validity of Short and Long Self-Administered Food Frequency Questionnaires in Ranking Dietary Intake in Middle-Aged and Elderly Japanese in the Japan Public Health Center-Based Prospective Study for the Next Generation (JPHC-NEXT) Protocol Area
Source: J Epidemiol. 2016 Aug 5;26(8):420–32. doi: 10.2188/jea.JE20150064 (PMC4967663; doi:10.2188/jea.JE20150064)
Supplement: Abstract in Japanese. [file je-26-420-s001.pdf]

詳細版食物摂取頻度調査票及びその短縮版による食物摂取状況推定の妥当性と比較－次世代多目的コホート研究 (JPHC-NEXT) プロトコル採用地域の中高年住民における検討－

横山悠太[1], 高地リベカ[1,2], 石原淳子[3], 石井有里[4], 笹月静[4], 澤田典絵[4],
篠澤友里恵[4], 田中純太[5], 加藤恵理香[1], 北村香織[1], 中村和利[1], 津金昌一郎[4]

1 新潟大学大学院医歯学総合研究科環境予防医学分野
2 奈良女子大学研究院生活環境科学系食物栄養学領域
3 相模女子大学栄養科学部管理栄養学科
4 国立がん研究センターがん予防・検診研究センター予防研究グループ
5 新潟大学大学院医歯学総合研究科健康増進医学講座

背景・目的：長期の前向きコホート研究では、追跡調査による摂取量情報の更新と同時に、被調査者の回答にかかる負担軽減も同時に要求される。我々は、次世代多目的コホート研究（JPHC-NEXT）ベースライン調査で、JPHC Study FFQ を基に開発した詳細版 FFQ（172 食品項目）を用いたが、追跡調査のために短縮版 FFQ（66 食品項目）を開発した。そこで、ベースラインで用いた短縮版及び詳細版 FFQ の順位づけ妥当性を当該調査対象の地域住民にて比較した。
方法：2012 年～2013 年に、JPHC-NEXT 詳細版 FFQ を使用する国内 5 地域の 40–74 歳の男女 240 人が 12 日間の秤量食事記録調査(12d-WFR)、詳細版 FFQ へ回答を完了した。そのうち 228 人は短縮版 FFQ も提出した。２種類の FFQ から計算した摂取量推定値と 12d-WFR との Spearman 相関係数を求め、個人内変動を補正した。
結果：短縮版 FFQ によるエネルギー及び 53 栄養素項目の推定値と 12d-WFR との相関係数の中央値は、男性で 0.46、女性で 0.44 であった。詳細版のそれはそれぞれ 0.50、0.43 であった。短縮版・詳細版 FFQ による栄養素等摂取量 5 分位による順位づけの、同分位か隣接分位内に一致した者の割合は、男性で 68%～91%、女性では 58%～85%であった。
まとめ：中高年の地域住民において、短縮版 FFQ でも、詳細版 FFQ と同様に多くの栄養素・食品群で概ね中程度以上に順位づけ可能な推定値が得られることが確認された。短縮版 FFQ は、前向き研究の追跡調査において食事情報の更新を目的として利用可能である。

キーワード：妥当性、食物摂取頻度調査、食事記録、日本人
